# Innovative Aesthetic and Functional Orthodontic Planning with Hard and Soft Tissue Analyses

**DOI:** 10.3390/jcm14134458

**Published:** 2025-06-23

**Authors:** Andra-Alexandra Stăncioiu, Alexandru Cătălin Motofelea, Anca Adriana Hușanu, Lorena Vasica, Adelina Popa, Riham Nagib, Camelia Szuhanek

**Affiliations:** 1 Orthodontic Research Center ORTHO-CENTER, Discipline of Orthodontics I, Faculty of Dental Medicine, “Victor Babes” University of Medicine and Pharmacy Timisoara, 9 No., Revolutiei Bv., 300041 Timisoara, Romania; andra.stancioiu@umft.ro (A.-A.S.); husanu.anca@umft.ro (A.A.H.); popa.adelina@umft.ro (A.P.); nagib.riham@umft.ro (R.N.); cameliaszuhanek@umft.ro (C.S.); 2 Dental Clinic Arad, Vicentiu Babes Sq., 310029 Arad, Romania; lorenavasica@yahoo.com; 3 Center for Molecular Research in Nephrology and Vascular Disease, Discipline of Nephrology, Department VII/Internal Medicine II, Faculty of Medicine, “Victor Babes” University of Medicine and Pharmacy, 300041 Timisoara, Romania

**Keywords:** AI, ANB, digital analysis, FMA, interincisal angle, L1 to LOP, lower lip to E-plane, nasolabial angle, orthodontics, U1 to UOP, upper lip to E-plane, WEBCEPH, Wits

## Abstract

**Background/Objectives:** This study aims to investigate the correlation between facial soft tissues, teeth, and skeletal growth patterns in order to provide an accurate diagnosis and orthodontic treatment plan using digital examination of lateral cephalograms. Achieving the best orthodontic outcome begins with an accurate, timely, and thorough diagnosis before starting the treatment phase. **Methods:** This cross-sectional study investigated the correlation between facial soft tissues, dental hard tissues, and skeletal growth patterns in 100 Romanian orthodontic patients (52 females, 48 males; median age 26 years) using digital lateral cephalograms. The measurements included skeletal parameters (ANB angle, FMA, Wits appraisal), dental parameters (inclinations of upper and lower incisors, interincisal angle), and soft tissue parameters (nasolabial angle, upper and lower lip positions relative to E-plane), all assessed using the AI-powered WEBCEPH software. Statistical analysis was conducted using RStudio (version 4.3.1). **Results:** A total of 100 subjects (52% female; median age, 26 years [range, 19–32 years]) were evaluated. No significant gender-based differences were found across cephalometric, soft tissue, or dental parameters. When stratified by ANB classification (Class I, 41%; Class II, 48%; Class III, 11%), significant differences emerged in the interincisal angle (*p* = 0.047), L1-to-LOP measurement (*p* < 0.001), lip-to-E-plane distances (*p* ≤ 0.009), Wits appraisal (*p* < 0.001), and the ANB angle itself (*p* < 0.001). Furthermore, stratification by FMA classification revealed expected differences in FMA values (*p* < 0.001) and a significant variation in the distribution of ANB classes (*p* = 0.042). All other cephalometric and soft tissue parameters remained comparable across FMA categories. **Conclusions:** The study highlights the importance of integrating hard and soft tissue analyses in orthodontic diagnosis and treatment planning to achieve optimal aesthetic and functional outcomes.

## 1. Introduction

The development of the dento-maxillary apparatus is a complex and prolonged process that may occasionally diverge from normal patterns. Morphological and functional abnormalities can arise from various developmental disorders affecting this system [1].

The skeletal and dental systems that support the varying thickness of facial soft tissues are essential to facial aesthetics. For a thorough orthodontic diagnosis and treatment plan in this social setting, it is crucial to ascertain the link between the exterior soft tissue and the underlying skeletal and dental hard tissue [2].

The technique that had the biggest impact on raising awareness of soft tissue as a diagnostic tool was cephalometric examination of soft profiles. Only recently did orthodontists acknowledge the functional and aesthetic aspects of the soft tissue as determinants in diagnosis and treatment planning, despite the fact that it was long emphasised that the soft-tissue facial profile may be just as important as the craniofacial skeleton in the assessment of orthodontic patients [3].

Numerous investigations have demonstrated the connection between facial characteristics and skeletal and occlusal alterations in malocclusions, coming to the conclusion that soft tissue examination can provide insight into skeletal and dental abnormalities. Consequently, facial analysis ought to be employed as a diagnostic method. Skeletal and soft tissues have been found to significantly correlate in the anteroposterior discrepancy [4,5,6,7].

Both hard and soft tissues create facial harmony, but soft tissue arrangement most impacts appearance. Soft tissue morphology is key for aesthetics, yet orthodontics research focuses on its skeletal pattern relationship. While individuality defines morphologies, soft tissue only partly reflects maxillomandibular relationships [8]. Cephalometry analyzes skeletal patterns, malocclusions, occlusal patterns, and compensations [8]. Soft tissue traits of occlusal patterns need separate examination, as hard tissue data does not indicate cutaneous correlations. Orthodontic treatment now emphasizes soft tissue improvements. Facial structures should be the main treatment objective, as balancing deficiencies affects their position. Long or short face patterns should guide soft tissue drape selection, and identifying compensations is crucial for optimal outcomes. Evaluating facial soft tissue and skeletal structure links appearance defects to dentoalveolar anatomy [8].

Dental specialties have been impacted by artificial intelligence, particularly in orthodontics through automated cephalometric analysis. Several AI-based cephalometric programs have proven reliable. Cephalometric analysis is vital for orthodontic diagnosis, treatment planning, and outcome evaluation. Manual cephalometric tracing, despite being the gold standard, is time-consuming and prone to errors. Automated cephalometric software was developed to address these limitations. These programs can perform analyses quickly once landmarks are plotted on digital cephalograms, with accuracy matching human experts. Artificial intelligence (AI) enables software to identify landmarks automatically. These automated programs significantly reduce orthodontists’ time in analyzing cases. Evaluating the accuracy of automatic landmark detection in software like WEBCEPH is crucial, as landmark identification remains a primary source of error in cephalometric analysis [9].

Numerous benefits of digital cephalometric examination include easier image acquisition, quicker measurements, easier sharing and archiving, quicker treatment planning, and fewer chemical dangers. Additionally, many analyses can be carried out simultaneously, and serial radiograph superimposition can be achieved more quickly [10,11].

Overall facial aesthetics are greatly influenced by the position of the incisors. Research indicates that some established standards of beauty are often regarded as desirable. These include the link between the incisors and other facial features, the grin arc, and lip support. Soft-tissue profile: An essential factor in deciding the incisor position is the interaction between the lips and the incisors. Lip balance and support can be affected by the incisor position, which can also affect the face’s overall appearance [12].

The aim of this study is to leverage AI-powered digital lateral cephalograms (WEBCEPH) to quantify how skeletal growth patterns (ANB, Wits, FMA) relate to dental inclinations (interincisal angle, incisor–occlusal plane), to measure soft-tissue profiles (upper and lower lip to the E-line, nasolabial angle), to identify the interdependencies among skeletal, dental, and soft-tissue parameters, and ultimately to support truly individualised orthodontic diagnosis and treatment planning.

## 2. Materials and Methods

The patients enrolled in the Discipline of Orthodontics I, Faculty of Dental Medicine, “Victor Babes” University of Medicine and Pharmacy, Timisoara, were the subjects of the study. All subjects provided written informed consent; the study was approved by the Institutional Ethics Committee of the “Victor Babes” University of Medicine and Pharmacy, Timisoara, Romania (CECS No. 04/26.01.2024).

A minimum of 100 patients were required for the sample. A prior study served as the basis for the effect size [13].

This cross-sectional study comprised 100 patients from Romania who visited the university clinic for orthodontic treatment to improve their dental and facial appearance and for a functional occlusion. A thorough clinical evaluation, including radiographs, scanning of the face and teeth, pre-treatment study models, and clinical photos, was performed on every patient. The inclusion and exclusion criteria were as follows: patients having high-quality x-ray pictures who did not have a syndromic cleft. Other than cleft lip and palate surgery, there was no history of craniofacial surgical treatment. There was no orthodontic treatment performed. There was a match with a healthy control who had no craniofacial abnormalities.

### 2.1. Procedure Methodology

#### 2.1.1. Digital Lateral Cephalometric Measurements and Protocol

As a reference, all lateral cephalograms of every patient were taken with their heads in their natural postures [14]. The participants were told to breathe through their nose and make contact with their molars during all lateral cephalograms, which were performed while they were standing erect and in their natural head position [15].

When measuring the angles and distances between skeletal landmarks as part of cephalometric analysis, precise head posture is crucial. However, a common operator error when obtaining lateral cephalograms is not placing individuals in the appropriate natural head posture (NHP) [16].

To reduce parallax distortion brought on by head movement, cephalometric radiography requires the use of a head holder, such as a cephalostat. The cephalostat guarantees consistent posture by supporting the patient’s head, which is essential for taking precise and repeatable measurements [17].

Qualified radiography technicians took lateral cephalograms using a PaX-i3D Green™ (city in the Republic of Korea city of Hwasong, Gyeonggi, VATECH) instrument. Scan time—scan ceph: 3.9 s, grayscale bit; the patient was standing or, if necessary, accessible in a wheelchair.

With the aid of AI and the Internet, WEBCEPH is an excellent web-powered orthodontic and orthognathic platform. For digital tracing, we employed the WEBCEPH—product: Dental Imaging Software; brand: WEBCEPH; model: WEBCEPH, Software Version: 2.0.0, AssembleCircle Corp., Seoul, Republic of Korea. The KFDA-approved and FDA 510(k)-cleared web-based orthodontic and orthognathic platform, WEBCEPHTM, is an AI-powered online orthodontic diagnostic tool for dentists. Both the Korean and US Intellectual Property Offices have granted patents to WEBCEPHTM’s AI inventions.

Each digital lateral cephalometric radiograph image was downloaded and stored on a Lenovo IdeaPad 5 Pro computer (manufacturer: Lenovo, series: IdeaPad 5 Pro 14IAP7, color: storm grey, form factor: laptop) prior to being imported into the WEBCEPH.

A free account was created in WEBCEPH, the lateral cephalograms were imported into this software then to the Digitization–Image Size Calibration section. After artificial intelligence automatically traced the cephalometric radiograph, we could use the “Modify” option to alter the landmark position, after which we saved the changes. Next, we navigated to the “Analysis” section, where we could select the type of analysis we wished to conduct; in this study, we chose the WEBCEPH analysis. From the menu, we could select the “View Mode”, which included line analysis, profilogram, and chart, among other options.

The lateral cephalogram images were 2256 × 2304 pixels in size, with 2256 pixels for the width and 2304 pixels for the height, 96 dpi for the horizontal and vertical resolution, and a bit depth of 24. WEBCEPH has been used by numerous studies to detect landmarks such as the following [18,19,20,21].

With the “Guide Ruler Size”, we specified the default size of the guide ruler for image size calibration—10 mm. With the “Pixels Per Millimeter for Cephalographs” option, we specified the default PPM (pixels per millimeter) for cephalographs—top of Form 5, 491 px/mm.

Table 1 shows the dental characteristics used in this study. First, the upper 1 to the upper occlusal plane was measured, which is abbreviated as U1 to UOP; it represents the U1–UOP angle between the upper occlusal plane and the upper incisor’s long axis. The lower 1 to lower occlusal plane, which is abbreviated L1 to LOP, represents the lower incisor inclination, that is, the angle created by the mandibular plane and the long axis of the lower incisor that is positioned most anteriorly, which was also measured. The angle separates the lower occlusal line from the lower incisor inclination line. Then, we also measured the interincisial angle, which is abbreviated as IIA, representing the angle that is obtuse between the upper and lower incisors’ long axes on the digital profile teleradiography.

Here we have extracted the mean values displayed in this WEBCEPH digital program: ANB—2.05° S.D. 1.8; FMA—25° S.D. 4.0; Wits appraisal—0.1 mm S.D. 1.9; upper lip to E-plane—4.7 mm S.D. 2.0; lower lip to E-plane—2 mm S.D. 2.0; nasolabial angle—95° S.D. 5.0.

**Figure 1:**
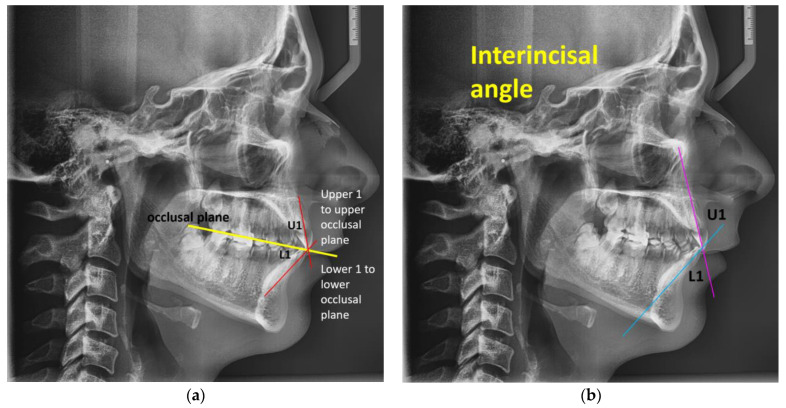
(**a**) Upper 1 to upper occlusal plane—U1 to UOP and lower 1 to lower occlusal plane—L1 to LOP. (**b**) Interincisal angle—IIA.

In Figure 1a, present the upper 1 to the upper occlusal plane, which is abbreviated as U1 to UOP, and the lower 1 to the lower occlusal plane, which is abbreviated as L1 to LOP. In Figure 1b, we present the interincisal angle that connects the upper incisor’s relative position to that of the lower incisor’s.

Anteroposterior Evaluation: ANB angle: The ANB angle examines the anteroposterior connection between the maxilla and mandible. The formula ANB = SNA − SNB is used to get the ANB angle, which is the difference between the SNA (sella–nasion to A-point) and SNB (sella–nasion to B-point) angles. The average ANB angle for a Class I skeletal pattern is 2 degrees. An ANB angle of more than 4 degrees reveals a Class II skeletal pattern, while an angle of less than 2 degrees indicates a Class III skeletal pattern. However, the prominence of the lower face and the location of the nasion can affect the ANB angle. When the ANB angle is abnormally increased or decreased, alternative methods such as the Wits analysis should be considered [24].

Sella—SELLA (S): The Turkish saddle’s (sella turcica) geometric center of outline; Nasion—NASION (N): The nose root is located at the most anterior position on the frontonasal suture in the mediosagittal plane; A-point: POINT A: the most posterior point on the anterior nasal spine’s curvature, in front of the upper central incisor root and beneath the SNA. B-point: POINT B is the farthest back on the mandibular alveolar process profile between Id and Pog, and the bone contour’s most posterior position between SNA and Pr [23].

The maxilla’s anteroposterior location in relation to the anterior cranial base is evaluated by the SNA angle. The SNA angle is made by joining the sella, nasion, and A-point. The average SNA angle is 81 ± 3 degrees. A patient who exhibits a well-positioned maxilla in relation to the cranial base, for instance, has an SNA angle of 82 degrees. A higher SNA angle shows that the maxilla is positioned protrusively relative to the cranial base compared to the average. On the other hand, if the SNA angle is smaller than usual, it indicates that the maxilla is retruded in relation to the cranial base. SNB angle: The SNB angle evaluates the anteroposterior position of the mandible to the anterior cranial base.

The SNB angle is formed by connecting the sella, nasion, and B-point. The average SNB angle is 78 ± 3 degrees. When the SNB angle is higher than normal, it means that the mandible is positioned anteriorly in relation to the cranial base. Conversely, a decreased SNB angle shows that the mandible is retruded relative to the cranial base compared to the norm [24].

**Figure 2:**
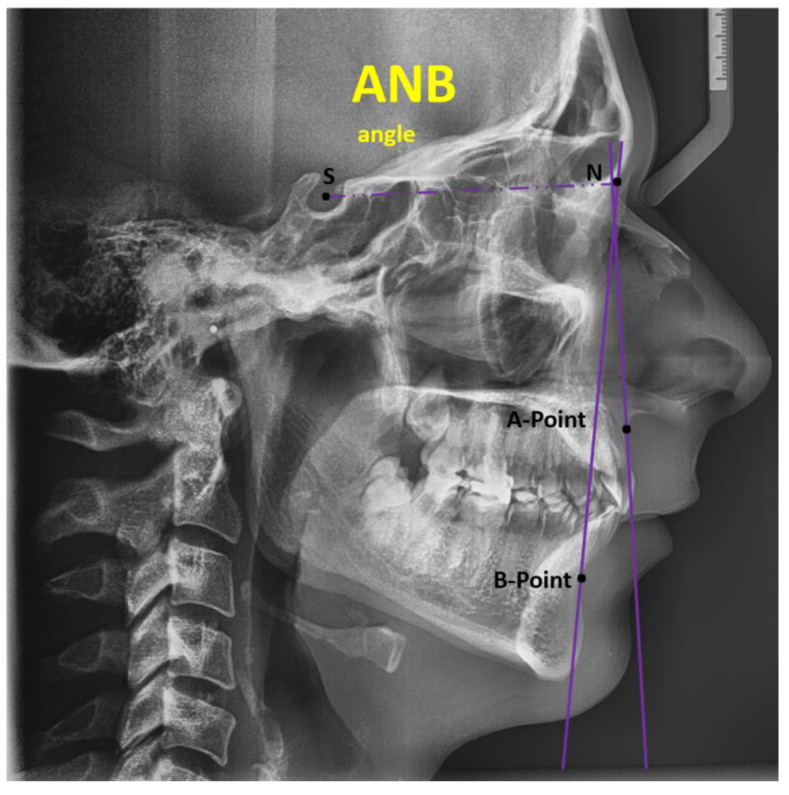
ANB angle formed by A-point, B-point, and Nasion.

Figure 2 shows the ANB angle, consisting of the A-point, B-point, and Nasion, which demonstrates the discrepancy between the maxilla and mandible in the sagittal plane. The ANB value is calculated as SNA-SNB.

Analysis of Wits: The Wits analysis provides an alternative approach to evaluating the anteroposterior skeletal pattern without relying on the cranial base. This method entails drawing perpendicular lines from locations A and B to the occlusal plane, defined as the line uniting the cusps of the posterior teeth. Points AO and BO are defined by the intersections of the occlusal plane with the perpendiculars from points A and B, respectively. The distance between AO and BO is then measured. For a class I skeletal pattern, BO is typically 1 mm (±1.9 mm) anterior to AO in males, while in females, BO and AO are generally equal (±1.77 mm) [24].

Ahmed M. et al., ANB angle: the angle formed by point A, Nasion, and point B (normal range = 0° to 4°). Wits appraisal: the linear distance between AO and BO (perpendicular drawn from points A and B onto the functional occlusal plane) (normal range = −1 mm to +1 mm) [25].

Wits: Cl. I for skeletal occlusion is −1 to +2 mm. Cl. II for skeletal occlusion is +3 mm and more. Cl. III for skeletal occlusion = −2 mm and less [23].

**Figure 3:**
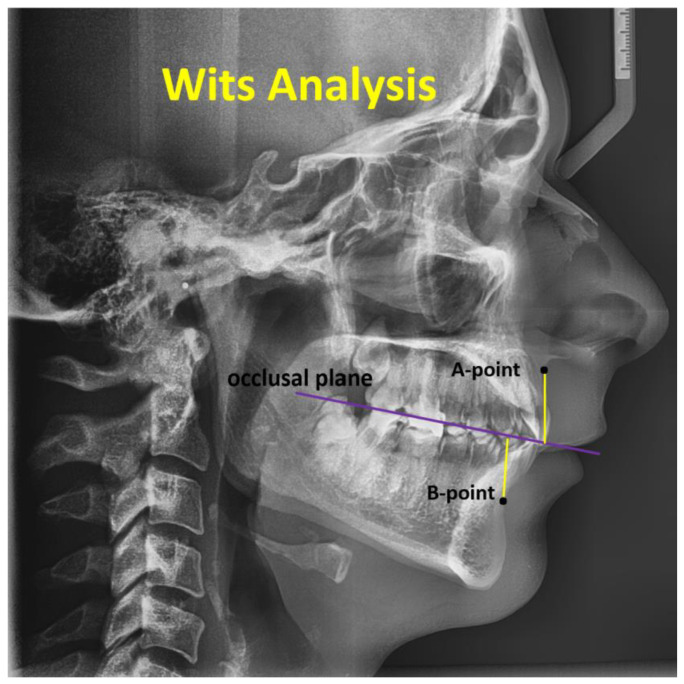
The Wits analysis is formed by the A-point, B-point, and occlusal plane.

Figure 3 displays the Wits analysis. The simple Wits appraisal of the jaw disharmony method can be used to assess the degree or severity of anteroposterior jaw dysplasia utilizing a lateral cephalometric film. It explains the maxilla’s relative location in the midsagittal plane in relation to the mandible. It is acquired by measuring the separation between the intersection points on the occlusal plane of two vertical lines drawn from locations A and B. A negative value is the outcome when point B is in front of point A.

Vertical evaluation: When the mandibular plane and the Frankfort horizontal plane connect, an angle known, as the FMA, is created. A diagnostic overlay can be used to measure and track this angle. Tweed deduced from clinical studies that the FMA typically varies between 16 and 35 degrees, with an average angle of 25 degrees. There are racial differences from this average, as well as possible differences by age and sex. Orthodontists utilize the general rule that an FMA of 25 degrees falls within the normal range. Patients with an FMA of 30 degrees or more are considered “high-angle,” whereas those with an FMA of 20 degrees or less are considered “low-angle.” In terms of vertical face types, open-bite skeletal patterns indicate a high FMA, while closed-bite skeletal patterns indicate a low FMA. It is important to distinguish between open-bite and closed-bite dental patterns and skeletal patterns. As a vital component of everyday treatment, a vast amount of data regarding growth and development, facial and bony traits, and other clinical manifestations at the extremities of the FMA (vertical facial types) defined by orthodontists is currently utilised. Generally speaking, alveolar bone development increases with a high FMA and decreases with a low FMA. Patients with low angles usually have high muscle attachments, small buccal vestibules, and flat, broad palate vaults. The converse is true for patients with high FMA. For a person with a low FMA, stability and retention could be issues. A skeletal deep bite and a less convex facial profile are characteristics of brachycephalic (short head) patients with a low FMA. An everted lower lip and a deep labiomental sulcus are common outcomes of mandibular overclosure. The presentation of teeth and gingiva is limited by the appearance of the top lip being lengthy. Lack of incisal contact causes high eruption and a deep vertical overlap of the anterior teeth, which frequently accentuates the curve of Spee. In order to determine the average Frankfort–mandibular plane angle, the current investigation was carried out [26].

Other values and characteristics of the FMA angle: it makes it possible to appreciate the vertical sense of the skeleton typology. This is usually 25.3°. Normodivergent growth occurs when the value is within normal limits. When the value surpasses 28°, the growth type is considered hyperdivergent. A score below 22° indicates a hypodivergent development type [23].

A component of the Tweed analysis is the FMA angle. The method’s foundation is the “TWEED’s triangle” that is created by assembling the following: Frankfurt plane: Porion–Orbital and mandibular plane: Gonion–Menton. The tip of the lower incisors and the incisal edge are connected by their axis [23].

**Figure 4:**
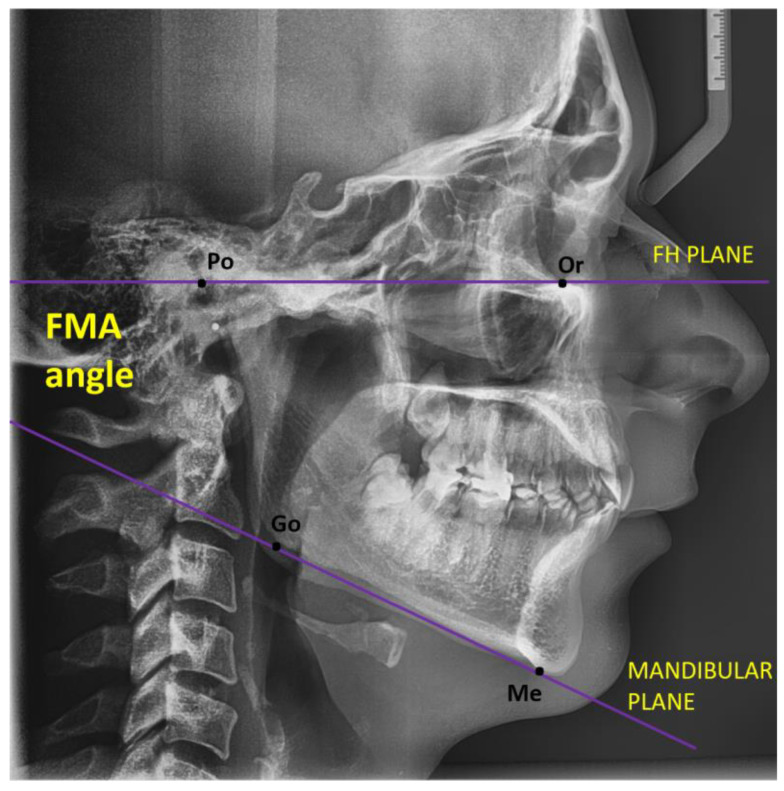
FMA angle composed from the FH plane and mandibular plane.

The following Figure 4 shows the FMA angle, which is made up of the Frankfort plane (made up of Porion–Orbital–Po–Or) and the mandibular plane (made up of Gonion–Menton–Go–Me).

Cutaneous landmarks evaluation (aesthetic analysis): Tangents to the columella and upper lip combine to generate the nasolabial angle. 102° is the usual value. The interpretation of labial retrusion is caused by elevated values. Low numbers indicate labial protrusion. Upper lip at line E (Pn–Pog)—Normal values: −4 ± 2 mm. Meaning: Elevated levels mean protrusion of the upper labial; low levels indicate retrusion of the upper labial region. Lower lip at line E (Pn–Pog)—Typical values are 2 ± 2 mm. Meaning: Higher values correspond to labial protrusion. Low readings are indicative of labial retrusion [23].

PRONASALE (Pn): The nose’s most anterior point, a unilateral skin point. Cutaneous point, unilateral cutaneous point POGONION (Pog) is the most anterior point of the chin in the mediosagittal plane [23].

**Figure 5:**
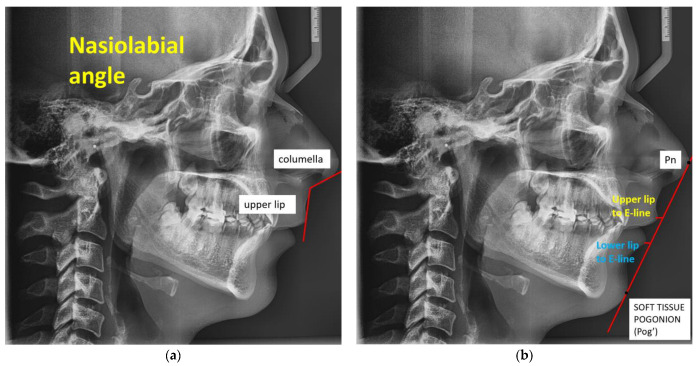
(**a**) Nasolabial angle formed by columella and upper lip; (**b**) upper lip at line E (Pn–Pog)(Pn—Pronasale; Pog—Pogonion) and lower lip at line E (Pn-Pog) (Pn—Pronasale; Pog—Pogonion).

In Figure 5a, we have the nasolabial angle, which is created by tangents to the upper lip and columella, and at Figure 5b, we have the upper lip at line E (Pn–Pog)(Pn—Pronasale; Pog—Pogonion) and the lower lip at line E (Pn–Pog), (Pn—Pronasale; Pog—Pogonion).

**Figure 6:**
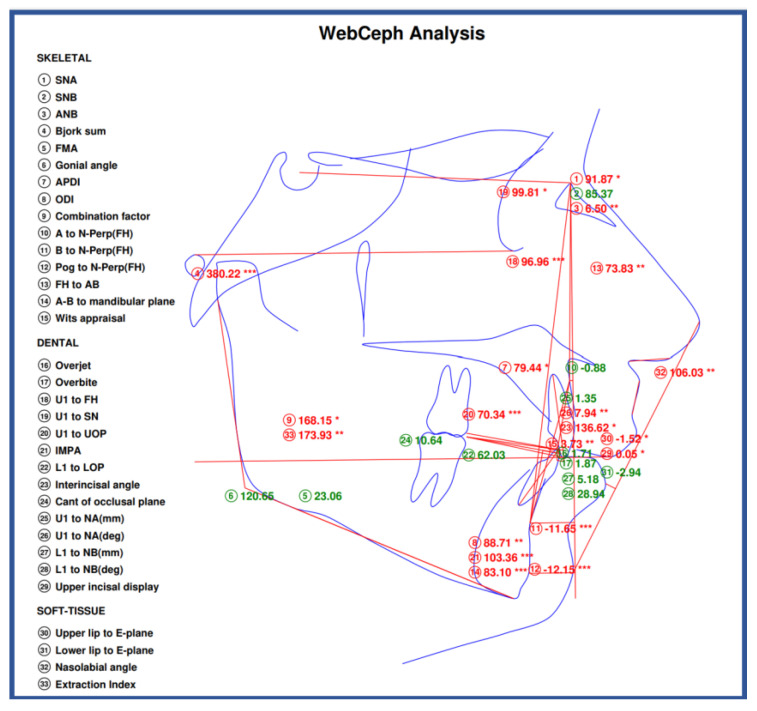
WEBCEPH Orthodontic Analysis Report. The sign “*” represents severity; a single sign “*” denotes the lowest severity, while multiple signs “*” denote a greater severity from normal values.

In Figure 6, we have exported an image directly from the WEBCEPH digital software, ‘’WEBCEPH Orthodontic Analysis Report,’’ with the measurements and parameters that are otherwise used in this study.

**Figure 7:**
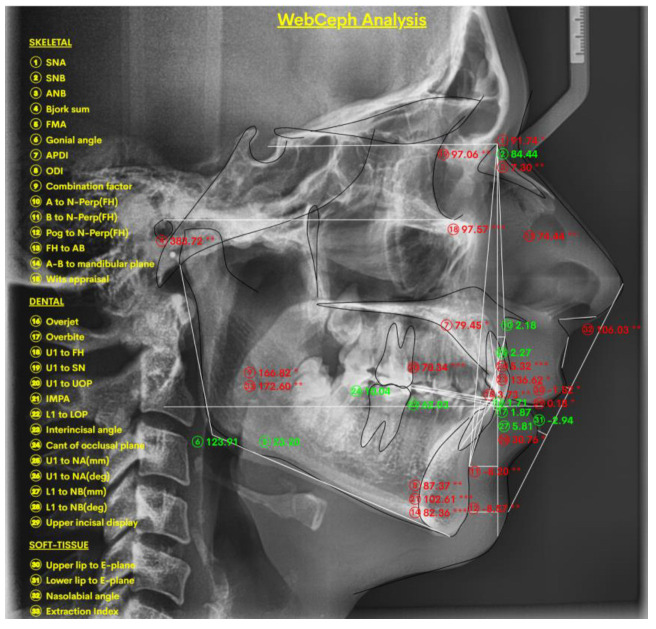
The digital lateral cephalogram in the WEBCEPH program is represented in View Mode, Line Analysis. The sign “*” represents severity; a single sign “*” denotes the lowest severity, while multiple signs “*” denote a greater severity from normal values.

In Figure 7, we present the lateral cephalogram entered in the WEBCEPH software in View Mode, Line Analysis (there is also a Profilogram and a Chart option), in which the Tracing Line, Analysis Line, and Measurements options allow users to change the colors in the software as desired so that they can be observed better.

**Figure 8:**
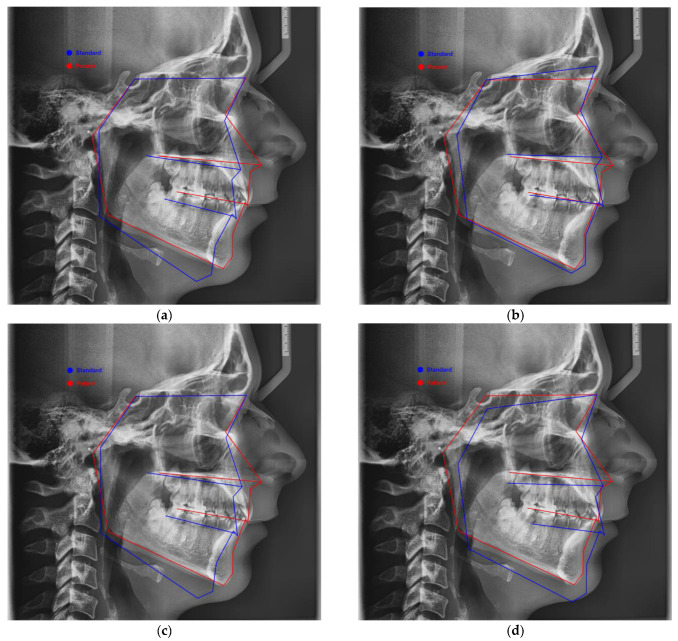
WEBCEPH: View Mode: Profilogram—(**a**) Origin: Sella and Plane: SN, (**b**) Origin: Sella and Plane: FH, (**c**) Origin: Nasion and Plane: SN, and (**d**) Origin: Nasion and Plane: FH.

The images presented in Figure 8 were taken from WEBCEPH, where we represented View Mode, Profilogram, in which (a) Origin: Sella and Plane: SN, (b) Origin: Sella and Plane: FH, (c) Origin: Nasion and Plane: SN, and (d) Origin: Nasion and Plane: FH, respectively. The blue line shows the standard, and the red line shows how the patient is. The horizontal lines represent, from top to bottom, the sella–nasion line, the palatal plane, the occlusal plane, and below it the mandibular plane.

In this study, and as a rule, we normally use the values of the parameters that are described in this book as well [23].

This study’s cephalometric parameter selection and interpretation are based on traditional analytical frameworks developed by Björk and Jarabak. Understanding craniofacial growth, mandibular rotation, and vertical and sagittal skeletal relationships—principles that are still essential in contemporary orthodontic diagnosis and treatment planning—was made possible by their groundbreaking work in the 20th century [27,28].

WEBCEPH is software that has been approved by the FDA and KFDA and has a proven track record of accuracy in automatic landmark detection. A study comparing WEBCEPH’S linear and angular cephalometric measurements with hand tracings was carried out by Mahto et al. [9] in 2022. According to their findings, there was good to excellent agreement between the two approaches, with all measurements showing intraclass correlation coefficient (ICC) values above 0.75 and many parameters exceeding 0.90. To evaluate intraobserver reliability, 20% of the photos in our study were reanalyzed by the same examiner three weeks later. ICC values >0.75, which indicate good consistency, were obtained. To further support WEBCEPH’s application in clinical decision-making, future studies should directly compare its outputs with those of more conventional manual techniques.

#### 2.1.2. Statistical Analysis

Data entry was performed in Microsoft Excel, and the dataset was subsequently analyzed using RStudio (version 4.3.1). The primary aims of the statistical analysis were to assess the relationships among skeletal, dental, and soft-tissue measurements; evaluate differences based on demographic subgroups such as gender and age; and compare cephalometric parameters across different skeletal classifications (Wits, ANB, and FMA).

Prior to conducting formal analyses, each continuous variable was tested for normality using the Shapiro–Wilk test. The outcomes of these tests guided the choice of subsequent statistical procedures. An a priori sample size calculation was also performed based on the difference in the mandibular foramen–sigmoid notch distance observed between male (21.9 mm) and female (20.2 mm) subjects, with a pooled standard deviation of 2.85 mm. This calculation resulted in a Cohen’s d effect size of approximately 0.60, which represents a medium effect. With an assumed significance level (alpha) of 0.05 and a male-to-female ratio of 61:39, a minimum sample size of 95 participants was deemed necessary. Therefore, our final sample of 100 subjects was considered adequately powered for detecting medium-sized differences.

Comparisons by gender were conducted using an Independent Samples *t*-test for variables that were normally distributed, and for those that did not meet the normality assumption, the Mann–Whitney U test was applied. A One-Way Analysis of Variance (ANOVA) was used to assess differences in cephalometric measurements across these age groups; when Levene’s test indicated unequal variances, Welch’s ANOVA was employed. If overall group differences were significant, pairwise comparisons were conducted using Tukey’s Honestly Significant Difference (HSD) post hoc test.

Comparable procedures were followed for subgroup analyses based on skeletal classifications (Wits, ANB, and FMA). In each instance, the choice between parametric or non-parametric tests was driven by the distribution of the data for the variable being examined.

To evaluate the reproducibility of cephalometric landmark identification and the corresponding measurements, 20% of the images were re-evaluated by the same examiner after a three-week interval. The resulting repeated measures were compared using the Intraclass Correlation Coefficient (ICC), with ICC values greater than 0.75 considered indicative of acceptable intraobserver reliability.

All statistical tests were conducted as two-tailed analyses at a significance level of 0.05. In addition to providing *p*-values, effect sizes were reported where applicable to aid in the interpretation of the clinical relevance of the findings. Although multiple comparisons were inherent in the subgroup analyses, adjustments for multiple testing (such as those provided by Tukey’s HSD) were applied during post hoc evaluations in order to control for the risk of Type I error.

This refined statistical approach ensures robust and transparent analysis, supporting valid inferences regarding the interplay between facial skeletal, dental, and soft-tissue characteristics.

Intraclass Correlation Coefficient (ICC) values for the important cephalometric variables assessed during the intraobserver reliability test, together with their 95% confidence intervals, to improve transparency and facilitate repeatability. Among these are ICC = 0.91 for the ANB angle (95% CI: 0.83–0.96); 95% CI: 0.78–0.94; FMA: ICC = 0.88; 95% CI: 0.75–0.93; ICC = 0.86 for the upper lip to the E-plane; 95% CI: 0.73–0.91; and ICC = 0.84 for the lower lip to the E-plane.

## 3. Results

In this study, 100 subjects were analyzed. The demographic characteristics revealed a balanced distribution, with a median age of 26 years (range: 19–32 years) and 52 (52%) females. Cephalometric analysis revealed a median ANB angle of 4.53° (range: 2.65–7.00°), indicative of a predominantly skeletal Class I pattern with a slight tendency toward skeletal Class II in terms of the anteroposterior skeletal relationship. The median FMA was 22° (range: 16–26°), with 14 (14%) subjects classified as hyperdivergent, 46 (46%) as hypodivergent, and 40 (40%) as normodivergent. The Wits appraisal, with a median value of 2.1 mm (range: 0.0–4.3 mm), further supports the presence of moderate skeletal discrepancies, aligning closely with the ANB findings. Soft tissue measurements also provided critical insights; the upper lip-to-E-plane distance was −2.75 mm (range: from −4.86 to −1.30 mm) and the lower lip-to-E-plane distance was −2.3 mm (range: from −3.9 to −0.6 mm), while the nasolabial angle had a median of 101° (range: 97–110°), indicating a well-balanced facial profile. Dental relationships were meticulously evaluated by analyzing incisor positions relative to their respective occlusal planes. The upper incisors-to-upper occlusal plane (U1–UOP) measurement demonstrated a median of 59° (range: 55–64°), and the lower incisors-to-lower occlusal plane (L1–LOP) measurement exhibited a median of 65° (range: 60–69°). Additionally, the interincisal angle, recorded at 128° (range: 122–137°), underscores a harmonious incisal relationship (Table 2).

**Figure 9:**
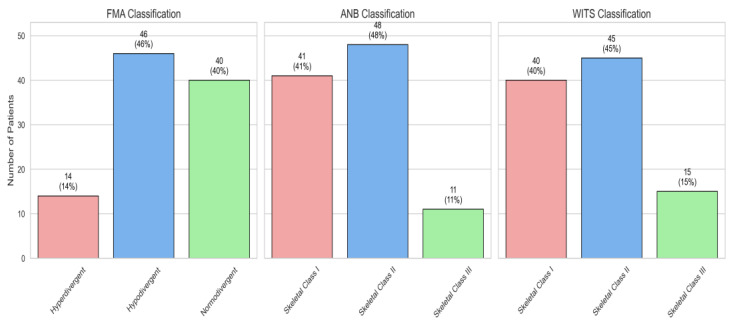
Distributions of the FMA, ANB, and WITS classifications across the sample size.

In Figure 9, we can observe a representation of the distributions of the FMA, ANB, and WITS classifications across the sample size as follows: FMA: 14% hyperdivergent, 46% hypodivergent, and 40% normodivergent. At ANB: 41% skeletal class I, 48% skeletal class II, and 11% skeletal class III. At WITS, 40% are in skeletal class I, 45% are in skeletal class II, and 15% are in skeletal class III.

In both genders, the median age was 26 years (females: IQR 20.5–32.0; males: IQR 18.8–32.2; *p* = 0.671). The median ANB angle was 4.3° in females (IQR 2.6–6.9) versus 4.6° in males (IQR 3.2–6.9; *p* = 0.858), and the median FMA was 21.1° (IQR 15.8–26.0) in females compared to 22.7° (IQR 19.0–25.6) in males (*p* = 0.572). The WITS appraisal was 2.1 mm in both groups (*p* = 0.994). The soft tissue measurements were similar, with the upper lip-to-E-plane distance at −3.0 mm (IQR −4.8 to −1.6) in females and −2.7 mm (IQR −4.9 to −1.0) in males (*p* = 0.424); the lower lip-to-E-plane was −2.3 mm in both (*p* = 0.586); and the nasolabial angle was 102.6° (IQR 96.6–109.8) in females versus 101.1° (IQR 96.4–109.7) in males (*p* = 0.406). Dental parameters, including the U1–UOP (59.5° vs. 58.1°, *p* = 0.361), L1–LOP (63.8° vs. 66.4°, *p* = 0.183), and interincisal angle (129.0° vs. 127.7°, *p* = 0.422), were comparable between genders. Similarly, skeletal classifications based on FMA, ANB, and Wits demonstrated no significant differences between females and males (*p* = 0.298, 0.981, and 0.986, respectively) (Table 3).

Stratifying the 100 subjects by Wits classification (40% Class I, 45% Class II, and 15% Class III) revealed significant differences in several cephalometric parameters. The interincisal angle was significantly higher in Class III (median 137.4° [129.1–146.1°]) compared to Class I (128.8° [122.7–134.7°]) and Class II (125.8° [119.8–133.6°], *p* = 0.010). The L1 to LOP measurement also varied significantly (*p* < 0.001), with Class III subjects showing a higher median (76.6° [71.8–79.3°]) than those in Class II (61.3° [57.0–64.4°]) and Class I (66.3° [64.1–69.5°]). In contrast, the U1 to UOP parameter did not differ significantly among the groups (*p* = 0.507). Regarding soft tissue, the nasolabial angle was the highest in Class II (median 104.1° [98.8–114.0°]) compared to Class I (100.7° [92.4–104.1°]) and Class III (99.4° [92.0–102.7°], *p* = 0.010). The lower lip-to-E-plane distance was most negative in Class III (−3.5 mm [−7.8 to −2.3 mm]), whereas it was less negative in Class I (−1.9 mm [−3.2 to −0.7 mm]) and Class II (−2.2 mm [−3.5 to −0.1 mm], *p* = 0.039). Similarly, the upper lip-to-E-plane measurement was significantly more negative in Class III (−5.5 mm [−7.6 to −4.4 mm]) than in Class I (−2.7 mm [−4.4 to −1.6 mm]) and Class II (−1.9 mm [−4.0 to −1.0 mm], *p* = 0.001). As expected, the Wits appraisal differed markedly across groups (*p* < 0.001), with medians of 1.0 mm (Class I), 4.5 mm (Class II), and −3.8 mm (Class III). Although the FMA values were comparable among the groups (*p* = 0.416), the ANB angle differed significantly (*p* < 0.001), with medians of 3.9° in Class I, 6.8° in Class II, and −0.2° in Class III. Furthermore, the distribution of ANB-based skeletal classifications varied significantly across the WITS groups (*p* < 0.001), with 70.0% of Class I, 80.0% of Class II, and 60.0% of Class III subjects assigned to their respective ANB classes. (Table 4)

Table 5 shows the subjects stratified into three groups based on FMA classification: hyperdivergent (n = 14, 14.0%), hypodivergent (n = 46, 46.0%), and normodivergent (n = 40, 40.0%). The interincisal angle, L1 to LOP, U1 to UOP, nasolabial angle, lower lip-to-E-plane, upper lip-to-E-plane, WITS appraisal, and ANB angle did not differ significantly among these groups, with *p*-values of 0.655, 0.230, 0.478, 0.423, 0.147, 0.713, 0.708, and 0.123, respectively. In contrast, the FMA measurement itself showed a highly significant difference (*p* < 0.001), with median values of 32.1° in the hyperdivergent group, 15.9° in the hypodivergent group, and 24.5° in the normodivergent group, which is expected given that these groups are defined by their FMA values. Moreover, while the continuous ANB values were similar across groups, the categorical distribution of ANB classifications revealed a significant difference (*p* = 0.042), suggesting that the pattern of skeletal sagittal discrepancies varies with the vertical growth pattern. Overall, aside from the inherent differences in FMA and the related categorical differences in ANB, the other cephalometric and soft tissue parameters remained largely comparable across the different vertical skeletal patterns.

In this analysis stratified by ANB classification, 100 subjects were divided into three groups: ANB Skeletal Class I (n = 41, 41.0%), Class II (n = 48, 48.0%), and Class III (n = 11, 11.0%). The interincisal angle differed significantly among groups (*p* = 0.047), with medians of 129.6° (IQR: 123.2–137.8°) in Class I, 125.7° (IQR: 120.7–132.8°) in Class II, and 130.0° (IQR: 126.0–146.1°) in Class III, indicating a modest but significant variation in dental incisal relationships. The L1 to LOP measurement also varied markedly (*p* < 0.001): subjects in Class I had a median of 66.3° (IQR: 63.8–70.3°), those in Class II had 61.4° (IQR: 57.3–65.3°), while Class III subjects recorded the highest median at 73.5° (IQR: 69.9–78.0°). By contrast, the U1 to UOP parameter remained statistically similar across groups (*p* = 0.873), with median values around 59° in each category.

Although the nasolabial angle did not differ significantly among the groups (*p* = 0.202), significant differences emerged in soft tissue lip positioning. The lower lip-to-E-plane measurement was −2.9 mm (IQR: −4.1 to −1.6 mm) in Class I, −1.3 mm (IQR: −2.7 to 0.6 mm) in Class II, and −3.3 mm (IQR: −7.1 to −0.4 mm) in Class III (*p* = 0.009). Similarly, the upper lip-to-E-plane distance varied significantly (*p* < 0.001): medians were −4.0 mm (IQR: −5.4 to −2.1 mm) in Class I, −1.8 mm (IQR: −3.1 to −0.5 mm) in Class II, and −5.4 mm (IQR: −7.6 to −2.6 mm) in Class III.

The WITS appraisal, a key indicator of anteroposterior skeletal relationships, also differed significantly (*p* < 0.001): Class I subjects had a median of 1.0 mm (IQR: −0.1 to 2.2 mm), Class II had 4.0 mm (IQR: 2.8 to 6.7 mm), and Class III had −2.7 mm (IQR: −4.2 to −2.3 mm). The FMA values demonstrated significant variability (*p* = 0.020), with median values of 19.0° (IQR: 15.2–24.5°) in Class I, 24.2° (IQR: 18.9–27.3°) in Class II, and 22.9° (IQR: 22.0–25.9°) in Class III. As expected, the ANB angle itself differed significantly across the groups (*p* < 0.001); the medians were 3.3° (IQR: 2.2–4.2°) for Class I, 7.0° (IQR: 5.7–8.1°) for Class II, and −0.3° (IQR: −0.9° to 0.1°) for Class III.

Furthermore, the categorical distribution of WITS classifications was significantly different (*p* < 0.001), with 68.3% of Class I subjects classified as WITS Skeletal Class I, 75.0% of Class II as WITS Skeletal Class II, and 81.8% of Class III as WITS Skeletal Class III. Similarly, the distribution of FMA classifications varied significantly among the ANB groups (*p* = 0.042).

Significant differences were observed in L1 to LOP, lip-to-E-plane measurements, WITS appraisal, FMA, and ANB angles across the ANB classifications, whereas U1 to UOP and nasolabial angle remained comparable. These findings underscore distinct craniofacial patterns associated with each ANB group, suggesting that subjects in ANB Skeletal Class II exhibit a more pronounced anteroposterior discrepancy, whereas those in Class III display a markedly different incisal and soft tissue profile (Table 6).

## 4. Discussion

This study examined the skeletal growth patterns, dental hard tissues, and facial soft tissues in 100 Romanian adults using comprehensive digital lateral cephalometric evaluations. ANB angle, FMA, and WITS assessment were the three basic cephalometric indices, and there were no discernible gender-based disparities among the participants, whose median age was 26. However, differences that were statistically significant were found when stratification was performed by skeletal classifications (WITS, FMA, and ANB). WITS skeletal Class III subjects showed higher L1 to LOP values, larger lip-to-E-plane distance deviations, and significant differences in interincisal and ANB angles compared to Class I and II subjects. The complex connection between dental orientation and underlying skeletal anatomy is further supported by these findings.

One of the key goals for patients receiving orthodontic treatment is still to achieve facial cosmetic harmony. It is still difficult to strike a balance between desired facial proportions and functional occlusion. The harmony of the soft-tissue envelope, which covers the facial bones and dentition, is essential because the face reflects both physical structure and social perception. The soft-tissue profile, skeletal linkages, and tooth location are all impacted by orthodontic, orthopedic, and orthognathic interventions. Both diagnosis and treatment planning depend on this interrelated structure. The transition to considering soft-tissue analysis and functional occlusion as key paradigms in contemporary orthodontics was summed up by Ackerman et al. [12].

Farha et al. [29] used relaxed lip posture in their cephalometric investigations to improve measurement accuracy, which is consistent with our methods. ANB angle and vertical patterns via FMA angle (ANB—Class I: 0–4°, Class II: >4°) were among the important parameters of their digital evaluation that we also evaluated [23]. The FMA threshold values utilised for vertical skeletal types (hypodivergent: <22°, normodivergent: 22–28°, hyperdivergent: >28°) are consistent with our classification.

The soft tissue profile of the ANB Skeletal Class III participants in our study was retrusive, with more negative upper and lower lip positions in comparison to the E-plane. This pattern is indicative of a more concave face profile, which is frequently regarded as less aesthetically pleasing. These observations align with those of Farha et al. [29], who observed that Class III profiles had greater nasolabial concavity and lip retrusion. Soft tissue compensation, such as incisor retroclination or lip weakening, may result from such skeletal abnormalities. Orthognathic surgery is used for more severe discrepancies, while dentoalveolar compensation is used for milder situations. E-plane distances should therefore be a key component of therapy diagnostics.

The soft-tissue cephalometric measures are shown here specifically. E-plane upper lip: According to Farha et al. [29], women’s results were −4.43 ± 2.04 mm and men’s were −5.34 ± 2.8 mm. Compared to Farha’s group, ours showed reduced upper lip retrusion, with a median value of −2.75 mm and an interquartile range (IQR) of −4.86 to −1.30 mm. E-plane lower lip: According to Farha et al., women’s results were −1.70 ± 2.5 mm and men’s were −2.85 ± 3.2 mm. Our analysis revealed a median of −2.3 mm with an IQR of −3.9 to −0.6 mm, which is in the same range but has a little less fluctuation. The angle of the nose: According to Farha et al., women’s angles ranged from 97.71° to 102.61°, while men’s angles ranged from 95.1° to 105.19°. With an IQR of 97° to 110°, our study’s median nasolabial angle was 101°, indicating a much broader range of variability but a similar central tendency.

In comparison to the esthetic plane, retrusion is indicated by negative lip-to-E-plane values. Measurement format differences could be a reflection of methodological or demographic differences between the studies.

Equally important is the function of vertical skeletal patterning. Long-faced people generally had higher incisal show, lip protrusion, and obtuse nasolabial angles, according to Jeelani et al. [30]; these features were largely replicated in our WITS and ANB Class III cases. According to Jeelani et al. [30], enhanced lip retrusion in Class III patients may have an impact on profile attractiveness and perceived age. These characteristics highlight how important it is to incorporate soft tissue evaluation into the planning of skeletal treatment. Strategies to restore facial balance may include incisor proclination or maxillary advancement, depending on the severity of the situation.

A fundamental understanding of vertical facial proportions can be gained from Da Vinci’s traditional concept of the “rule of thirds” [31]. This was further developed in more recent research by Abdul-Qadir et al. [32] and others [33,34] who connected soft tissue characteristics with vertical development. For example, I’erovic et al. [34] investigated how soft tissue thickness varied by gender across malocclusion classes.

As demonstrated by [2], the skeletal and dental structure has a significant impact on facial attractiveness. Soft tissue profiles, which were formerly overlooked in favor of skeletal and dental relationships, are now given a lot of weight in treatment planning due to changing orthodontic paradigms. Although Sassouni’s [35] classification system based on vertical skeletal dimensions was fundamental, recent studies have shown that soft tissue plays a significant influence in determining the final aesthetic result.

As a result, instruments for cephalometric analysis have changed. The conventional linear and angular approaches with reference planes [29,36] have been replaced by more advanced evaluations. There is increasing agreement in orthodontics and related fields, such as plastic surgery, regarding the importance of soft tissue profiles in determining facial harmony [37,38,39,40,41]. We followed Arnett and Gunson’s [42] recommendation to assess patients in their resting posture in order to record their soft tissue properties.

Other works have reflected this methodological consistency [43,44,45]. In accordance with standard practices, we excluded participants who had received substantial treatments in the past or who were younger than 18 years old in order to ensure reliable measurements [46].

Actionable insights are provided by notable intergroup discrepancies in our dataset, especially between WITS and ANB classifications. Class III groups’ retruded lip profiles and accentuated dental inclination highlight the need to combine dental compensation and skeletal realignment to improve aesthetics. Our results corroborate studies that demonstrate how incisor location, however minor, has a considerable impact on perceived facial balance and lip-to-E-plane distances.

Soft tissue morphology and skeleton class were shown to be strongly correlated. According to existing research, adolescents’ soft tissue profiles can be predicted with approximately 50% accuracy using anterior dental and skeletal markers [47], and these dimensions are further influenced by age and sex [47,48]. Such relationships were investigated in Saudi populations by Shamlan and Aldrees [49], who confirmed that hard tissue landmarks can predict related soft tissue features.

The use of the FDA- and KFDA-approved WEBCEPH platform demonstrated excellent intraobserver repeatability in our hands—mirroring findings by Mahto et al. [9] and Shamlan and Aldrees [49]—suggesting that AI-driven cephalometry can enhance both efficiency and consistency in routine diagnostics.

Likewise, the association between ANB and WITS assessments was validated by Santiago et al. [50]. In accordance with other frameworks, our classification scheme (Class I: ANB 0–4°; Class II: ANB > 4°; Class III: ANB < 0°) is appropriate [51]. Similarly to our findings, Shrestha et al. [52] found characteristics in Class II patients, including convex profiles, upper incisor proclination, and retrusive mandibles. Additionally, these results confirm that soft tissue patterns are applicable to a variety of groups.

Consistent with our findings, Jazmati et al. [53] used CBCT to measure soft tissue thickness and found distinctive lower facial features in Class III patients. In Class III individuals, Kale et al. [54] also noted large chins and decreased upper lip volume, establishing a connection between soft tissue presentation and skeletal structure. These revelations highlight the necessity of both bone and soft tissue examination in thorough orthodontic evaluations. While dental camouflage, such as incisor proclination, may be helpful in borderline situations, orthodontic surgery is still a crucial option for severe cases. Meaningful subgroup analyses by skeletal class and sex are made possible by the inclusion of 100 untreated orthodontic patients (52 girls and 48 men), an approach that was frequently absent from earlier studies with smaller or gender-skewed samples [55].

Our study adds more accuracy and breadth to existing frameworks without putting out a whole new paradigm. An important step toward customised, aesthetically pleasing orthodontic treatment is the combination of hard and soft tissue assessment with AI-powered cephalometric technologies. Our results confirm the important interaction of soft tissue shapes, dental alignment, and skeletal architecture in effective treatment planning. In the end, a more sophisticated understanding of facial equilibrium is offered by orthodontic diagnostics that combine skeletal, dental, and soft tissue information. This all-encompassing strategy is in line with contemporary developments in clinical orthodontics that prioritize individualised treatment plans and superior aesthetics. As demonstrated by Tweed [56], the Björk–Jarabak study [28], and the dual-structure approach presented by Arnett and Bergman [57], our AI-based analytical platform was effective, repeatable, and clinically significant.

### Study Limitations and Future Perspectives

While our study provides a multidimensional analysis by incorporating demographic, cephalometric, and soft tissue variables, it is limited by its cross-sectional design and relatively modest sample size. The cross-sectional design of this study is one of its main limitations, as it limits the capacity to infer causal relationships from the connections that were found. To assess how skeletal, dental, and soft tissue parameters change over time and to gain a deeper understanding of the temporal interactions between them, longitudinal studies would be required. Future research would benefit from longitudinal studies that track how these parameters evolve with orthodontic treatment and growth, thereby further validating the prognostic value of combined hard–soft tissue analyses. The sample is comparatively homogeneous, consisting solely of Romanian individuals between the ages of 19 and 32. The results cannot be applied to larger, more varied populations with different age groups and ethnic origins because of this demographic constraint. To improve external validity, volunteers from various age groups, ethnic backgrounds, and geographical areas should be included in future research. Third, despite its continued use and accessibility as a diagnostic technique in orthodontics, two-dimensional (2D) lateral cephalometry has intrinsic drawbacks. Volumetric and transverse differences, which could be clinically significant in evaluating facial asymmetry or depth-related traits, are specifically not captured by 2D imaging. Incorporating three-dimensional (3D) imaging modalities, such as CBCT, into future research could improve diagnostic precision and treatment planning by offering more thorough insights into craniofacial morphology and spatial interactions. The study’s measurements were all taken by a single assessor. Although the evaluation of intraobserver reliability was deemed satisfactory, the lack of interobserver assessment could potentially induce bias. Several blinded evaluators should be used in future studies to improve methodological rigor even more.

## 5. Conclusions

Using AI-assisted digital cephalometric analysis using the WEBCEPH platform, this study offers a thorough assessment of the relationship between skeletal growth patterns, dental hard tissues, and facial soft tissues in an adult population from Romania. We found persistent and clinically significant changes in dental inclinations and lip positions by stratifying individuals across several skeletal categorisation systems, including ANB, WITS, and FMA. These discrepancies were especially noticeable among Class II and Class III skeletal patterns. Illustrating compensatory dental and soft tissue adaptations to skeletal discrepancies, Class III patients showed noticeably greater lower incisor proclination and more retruded lip profiles, while Class II patients showed the opposite trend. Our results demonstrate the diagnostic utility of integrating dental, soft tissue, and skeleton data in a multiparameter, organized manner. In addition to increasing productivity and reproducibility, the introduction of AI-driven digital tools made it possible to conduct sophisticated evaluations that conventional two-dimensional studies could otherwise miss. In contemporary orthodontic practice, where attaining facial harmony and customized treatment planning are given equal weight with occlusal correction, this integrated diagnostic concept is very pertinent.

## Figures and Tables

**Table 1:** Dental characteristics measured in the WEBCEPH digital software.

Variables	Short Form	Details
Upper 1 to upper occlusal plane	U1 to UOP	Angle between the upper occlusal plane and the upper incisor’s long axis [22]. Mean values from the WEBCEPH digital program: 55°, S.D. 4.0.
Lower 1 to lower occlusal plane	L1 to LOP	Angle between the lower occlusal plane and the lower incisor’s long axis [22]. Mean values from the WEBCEPH digital program: 66°, S.D. 5.0.
Interincisal angle	IIA	Angle formed by the upper and lower incisors’ long axes [22]. The mutual angulation between the incisors is expressed by the interincisal angle, which is independent of the bone bases. Value normal: 130 ± 5°. When the value exceeds 135°, the incisors are retroclined. When the value is less than 125°, the incisors are proclined [23]. Mean values from the WEBCEPH digital program: 130°, S.D. 5.8.
Occlusal plane	OP	Occlusal plane: the line that, in the event of open frontal occlusion, crosses the space between the central incisors or the midway point of the overbite to split the occlusion of the first molars; following Ricketts, a line that passes across the greatest number of points of contact between the superior and inferior ocular bones represents linear deformation caused by Spee [23].

**Table 2:** Summary of demographic, cephalometric, and classification data.

Characteristic	Value
Age (years)	26 (19, 32)
Gender	
Female	52 (52%)
Male	48 (48%)
ANB (degrees)	4.53 (2.65, 7.00)
FMA (degrees)	22 (16, 26)
Wits (mm)	2.1 (0.0, 4.3)
Upper lip to E-plane (mm)	−2.75 (−4.86, −1.30)
Lower lip to E-plane (mm)	−2.3 (−3.9, −0.6)
Nasolabial angle (degrees)	101 (97, 110)
U1 to UOP (upper incisors to upper occlusal plane)	59 (55, 64)
L1 to LOP (lower incisors to lower occlusal plane)	65 (60, 69)
Interincisal angle (degrees)	128 (122, 137)

Footer: The data are presented as median (Q1, Q3) for continuous variables and n (%) for categorical variables. Notes: ANB uses the following formula to determine the difference between the SNA (sella–nasion to A-point) and SNB (sella–nasion to B-point) angles: SNA minus SNB = ANB; FMA is created when the mandibular plane and the Frankfort horizontal plane cross. Abbreviations: Frankfort mandibular plane; Wits—Wits appraisal; U1–UOP—upper incisors to upper occlusal plane; L1–LOP—lower incisors to lower occlusal plane.

**Table 3:** Comparison of sex-dependent demographic, cephalometric, soft tissue, and dental parameters.

Dependent: Gender		Female	Male	Total	*p*
Total N (%)		52 (52.0)	48 (48.0)	100	
Age (years)	Median (IQR)	26.0 (20.5 to 32.0)	26.0 (18.8 to 32.2)	26.0 (19.0 to 32.0)	0.671
ANB degrees	Median (IQR)	4.3 (2.6 to 6.9)	4.6 (3.2 to 6.9)	4.5 (2.6 to 6.9)	0.858
FMA degrees	Median (IQR)	21.1 (15.8 to 26.0)	22.7 (19.0 to 25.6)	22.2 (16.5 to 25.7)	0.572
Wits (mm)	Median (IQR)	2.1 (−0.1 to 4.3)	2.1 (0.1 to 4.2)	2.1 (0.0 to 4.3)	0.994
Upper lip to E-plane (mm)	Median (IQR)	−3.0 (−4.8 to −1.6)	−2.7 (−4.9 to −1.0)	−2.8 (−4.8 to −1.3)	0.424
Lower lip to E-plane (mm)	Median (IQR)	−2.3 (−3.5 to −1.1)	−2.3 (−4.1 to −0.1)	−2.3 (−3.8 to −0.6)	0.586
Nasolabial angle	Median (IQR)	102.6 (96.6 to 109.8)	101.1 (96.4 to 109.7)	101.5 (96.5 to 109.7)	0.406
U1 to UOP	Median (IQR)	59.5 (56.6 to 63.9)	58.1 (53.8 to 62.5)	58.8 (54.9 to 63.8)	0.361
L1 to LOP	Median (IQR)	63.8 (59.0 to 68.0)	66.4 (61.2 to 69.7)	65.2 (60.5 to 69.4)	0.183
Interincisal angle	Median (IQR)	129.0 (123.0 to 137.9)	127.7 (121.0 to 135.7)	128.2 (121.6 to 136.8)	0.422
Meaning results of FMA	Hyperdivergent	8 (15.4)	6 (12.5)	14 (14.0)	0.298
	Hypodivergent	27 (51.9)	19 (39.6)	46 (46.0)	
	Normodivergent	17 (32.7)	23 (47.9)	40 (40.0)	
Meaning results of ANB	Skeletal Class I	21 (40.4)	20 (41.7)	41 (41.0)	0.981
	Skeletal Class II	25 (48.1)	23 (47.9)	48 (48.0)	
	Skeletal Class III	6 (11.5)	5 (10.4)	11 (11.0)	
Meaning results WITS	Skeletal Class I	21 (40.4)	19 (39.6)	40 (40.0)	0.986
	Skeletal Class II	23 (44.2)	22 (45.8)	45 (45.0)	
	Skeletal Class III	8 (15.4)	7 (14.6)	15 (15.0)	

**Table 4:** Cephalometric Parameters According to WITS Classification Groups.

Parameter	Meaning Results WITS Skeletal Class I	Meaning Results WITS Skeletal Class II	Meaning Results WITS Skeletal Class III	Total	*p*-Value
Total N (%)	40 (40.0)	45 (45.0)	15 (15.0)	100	
Interincisal angle (degrees)	128.8 (122.7 to 134.7)	125.8 (119.8 to 133.6)	137.4 (129.1 to 146.1)	128.2 (121.6 to 136.8)	0.01
L1 to LOP (degrees)	66.3 (64.1 to 69.5)	61.3 (57.0 to 64.4)	76.6 (71.8 to 79.3)	65.2 (60.5 to 69.4)	<0.001
U1 to UOP (degrees)	58.8 (54.5 to 60.8)	59.6 (55.7 to 64.5)	57.2 (52.2 to 65.5)	58.8 (54.9 to 63.8)	0.507
Nasolabial angle (degrees)	100.7 (92.4 to 104.1)	104.1 (98.8 to 114.0)	99.4 (92.0 to 102.7)	101.5 (96.5 to 109.7)	0.01
Lower lip to E-plane (mm)	−1.9 (−3.2 to −0.7)	−2.2 (−3.5 to −0.1)	−3.5 (−7.8 to −2.3)	−2.3 (−3.8 to −0.6)	0.039
Upper lip to E-plane (mm)	−2.7 (−4.4 to −1.6)	−1.9 (−4.0 to −1.0)	−5.5 (−7.6 to −4.4)	−2.8 (−4.8 to −1.3)	0.001
Wits (mm)	1.0 (0.0 to 2.0)	4.5 (3.5 to 7.0)	−3.8 (−4.4 to −2.6)	2.1 (0.0 to 4.3)	<0.001
FMA (degrees)	23.4 (15.8 to 27.4)	21.6 (16.3 to 24.4)	22.9 (18.8 to 24.8)	22.2 (16.5 to 25.7)	0.416
ANB (degrees)	3.9 (2.6 to 4.7)	6.8 (5.4 to 8.1)	−0.2 (−0.9 to 1.7)	4.5 (2.6 to 6.9)	<0.001
Meaning results of FMA					0.558
Hyperdivergent	8 (20.0)	5 (11.1)	1 (6.7)	14 (14.0)	
Hypodivergent	18 (45.0)	22 (48.9)	6 (40.0)	46 (46.0)	
Normodivergent	14 (35.0)	18 (40.0)	8 (53.3)	40 (40.0)	
Meaning results of ANB					<0.001
Skeletal Class I	28 (70.0)	8 (17.8)	5 (33.3)	41 (41.0)	
Skeletal Class II	11 (27.5)	36 (80.0)	1 (6.7)	48 (48.0)	
Skeletal Class III	1 (2.5)	1 (2.2)	9 (60.0)	11 (11.0)	

Footer: Continuous variables are expressed as median (interquartile range) and categorical variables as n (%). *p*-values represent the statistical significance of differences across WITS classification groups, with tests appropriate to the variable distribution. Abbreviations: L1–LOP—lower incisors to lower occlusal plane; U1–UOP—upper incisors to upper occlusal plane; WITS—Wits appraisal; FMA—Frankfort mandibular plane angle; ANB—A Point–Nasion–B Point angle.

**Table 5:** Cephalometric Parameters Stratified by FMA Classification (N = 100).

Parameter	Hyperdivergent	Hypodivergent	Normodivergent	Total	*p*-Value
Total N (%)	14 (14.0)	46 (46.0)	40 (40.0)	100	
Interincisal angle (°)	127.9 (121.6 to 133.5)	128.0 (120.1 to 136.8)	128.7 (123.0 to 137.9)	128.2 (121.6 to 136.8)	0.655
L1 to LOP (°)	63.8 (60.8 to 69.0)	64.7 (58.8 to 67.9)	66.1 (62.8 to 72.0)	65.2 (60.5 to 69.4)	0.230
U1 to UOP (°)	59.9 (57.0 to 61.8)	58.5 (53.2 to 61.8)	58.8 (55.6 to 64.6)	58.8 (54.9 to 63.8)	0.478
Nasolabial angle (°)	103.8 (100.3 to 109.4)	100.1 (94.5 to 108.8)	101.6 (97.0 to 109.7)	101.5 (96.5 to 109.7)	0.423
Lower lip to E-plane (mm)	−1.1 (−1.3 to 0.3)	−2.5 (−3.5 to −1.4)	−2.1 (−4.5 to −0.2)	−2.3 (−3.8 to −0.6)	0.147
Upper lip to E-plane (mm)	−2.4 (−3.3 to −0.8)	−3.0 (−4.6 to −1.6)	−2.7 (−5.4 to −1.3)	−2.8 (−4.8 to −1.3)	0.713
WITS (mm)	1.7 (0.1 to 5.1)	2.4 (0.5 to 4.3)	2.1 (−1.1 to 4.2)	2.1 (0.0 to 4.3)	0.708
FMA (°)	32.1 (30.9 to 34.7)	15.9 (14.5 to 19.0)	24.5 (23.4 to 26.1)	22.2 (16.5 to 25.7)	<0.001
ANB (°)	6.0 (3.6 to 7.9)	4.2 (2.5 to 5.8)	5.0 (2.3 to 7.3)	4.5 (2.6 to 6.9)	0.123
Meaning results of ANB					0.042
Skeletal Class I	4 (28.6)	26 (56.5)	11 (27.5)	41 (41.0)	
Skeletal Class II	9 (64.3)	17 (37.0)	22 (55.0)	48 (48.0)	
Skeletal Class III	1 (7.1)	3 (6.5)	7 (17.5)	11 (11.0)	
Meaning results of WITS					0.558
Skeletal Class I	8 (57.1)	18 (39.1)	14 (35.0)	40 (40.0)	
Skeletal Class II	5 (35.7)	22 (47.8)	18 (45.0)	45 (45.0)	
Skeletal Class III	1 (7.1)	6 (13.0)	8 (20.0)	15 (15.0)	

Footer: Continuous variables are presented as median (interquartile range) and categorical variables as n (%). *p*-values indicate the significance of differences across FMA classifications (hyperdivergent, hypodivergent, normodivergent) using appropriate statistical tests. Abbreviations: L1–LOP—lower incisors to lower occlusal plane; U1–UOP—upper incisors to upper occlusal plane; WITS—Wits appraisal; FMA—Frankfort–mandibular Plane Angle; ANB—A Point–Nasion–B Point angle.

**Table 6:** Cephalometric parameters stratified by ANB classification (N = 100).

Parameter	ANB Skeletal Class I (41, 41.0%)	ANB Skeletal Class II (48, 48.0%)	ANB Skeletal Class III (11, 11.0%)	Total	*p*-Value
Interincisal angle (°)	129.6 (123.2 to 137.8)	125.7 (120.7 to 132.8)	130.0 (126.0 to 146.1)	128.2 (121.6 to 136.8)	0.047
L1 to LOP (°)	66.3 (63.8 to 70.3)	61.4 (57.3 to 65.3)	73.5 (69.9 to 78.0)	65.2 (60.5 to 69.4)	<0.001
U1 to UOP (°)	59.0 (55.3 to 61.6)	58.9 (55.5 to 64.5)	57.2 (52.2 to 64.5)	58.8 (54.9 to 63.8)	0.873
Nasolabial angle (°)	101.5 (96.5 to 107.0)	102.6 (96.9 to 114.0)	99.0 (92.0 to 100.6)	101.5 (96.5 to 109.7)	0.202
Lower lip to E-plane (mm)	−2.9 (−4.1 to −1.6)	−1.3 (−2.7 to 0.6)	−3.3 (−7.1 to −0.4)	−2.3 (−3.8 to −0.6)	0.009
Upper lip to E-plane (mm)	−4.0 (−5.4 to −2.1)	−1.8 (−3.1 to −0.5)	−5.4 (−7.6 to −2.6)	−2.8 (−4.8 to −1.3)	<0.001
WITS (mm)	1.0 (−0.1 to 2.2)	4.0 (2.8 to 6.7)	−2.7 (−4.2 to −2.3)	2.1 (0.0 to 4.3)	<0.001
FMA (°)	19.0 (15.2 to 24.5)	24.2 (18.9 to 27.3)	22.9 (22.0 to 25.9)	22.2 (16.5 to 25.7)	0.020
ANB (°)	3.3 (2.2 to 4.2)	7.0 (5.7 to 8.1)	−0.3 (−0.9 to 0.1)	4.5 (2.6 to 6.9)	<0.001
Meaning results of WITS					<0.001
Skeletal Class I	28 (68.3)	11 (22.9)	1 (9.1)	40 (40.0)	
Skeletal Class II	8 (19.5)	36 (75.0)	1 (9.1)	45 (45.0)	
Skeletal Class III	5 (12.2)	1 (2.1)	9 (81.8)	15 (15.0)	
Meaning results of FMA					0.042
Hyperdivergent	4 (9.8)	9 (18.8)	1 (9.1)	14 (14.0)	
Hypodivergent	26 (63.4)	17 (35.4)	3 (27.3)	46 (46.0)	
Normodivergent	11 (26.8)	22 (45.8)	7 (63.6)	40 (40.0)	

Footer: Continuous variables are expressed as median (interquartile range) and categorical variables as n (%). *p*-values represent the statistical significance of differences across ANB classification groups (ANB—Skeletal Class I, II, and III) using appropriate statistical tests. Abbreviations: L1–LOP—lower incisors to lower occlusal plane; U1–UOP—upper incisors to upper occlusal plane; WITS—Wits appraisal; FMA—Frankfort mandibular plane angle; ANB—A Point–Nasion–B Point angle.

## Data Availability

All data regarding this manuscript can be requested from the corresponding author at alexandru.motofelea@umft.ro.
